# Reconsidering the prognosis of major depressive disorder across diagnostic boundaries: full recovery is the exception rather than the rule

**DOI:** 10.1186/s12916-017-0972-8

**Published:** 2017-12-12

**Authors:** Judith Verduijn, Josine E. Verhoeven, Yuri Milaneschi, Robert A. Schoevers, Albert M. van Hemert, Aartjan T. F. Beekman, Brenda W. J. H. Penninx

**Affiliations:** 1 Department of Psychiatry, Amsterdam Public Health Research Institute, VU University Medical Center, PO Box 74077, 1070 BB Amsterdam, The Netherlands; 2 Department of Psychiatry, University Medical Center Groningen, University of Groningen, Groningen, The Netherlands; 3 Department of Psychiatry, Leiden University Medical Center, Leiden, The Netherlands

**Keywords:** Major depressive disorder, Affective disorder, Anxiety disorder, Course, Prognosis, Longitudinal, Comorbidity

## Abstract

**Background:**

Major depressive disorder (MDD) is often handled as an episodic and isolated disorder, resulting in an optimistic view about its prognosis. Herein, we test the idea that the prognosis of MDD changes if we vary the perspective in terms of (1) a longer time frame and (2) a broader diagnostic conceptualisation including dysthymia, (hypo)mania and anxiety disorders as relevant outcomes.

**Methods:**

Patients with current MDD at baseline (*n* = 903) and available 2-, 4-, and/or 6-year follow-up assessments were selected from the Netherlands Study of Depression and Anxiety, a psychiatric cohort study. Combining psychiatric DSM-IV-based diagnoses and life-chart data, patient course trajectories were classified as (1) recovered (no diagnoses at 2-year follow-up or thereafter), (2) recurrent without chronic episodes, (3) recurrent with chronic episodes or (4) consistently chronic since baseline. A chronic episode was defined as having a current diagnosis at the follow-up assessment and consistent symptoms over 2 years. Proportions of course trajectories were provided moving from a short, narrow perspective (2-year follow-up, considering only MDD diagnosis) to a long, broad perspective (6-year follow-up, including MDD, dysthymia, (hypo)mania and anxiety diagnoses).

**Results:**

With the short, narrow perspective, the recovery rate was 58% and 21% had a chronic episode. However, in the long, broad perspective the recovery rate was reduced to 17%, while 55% of the patients experienced chronic episodes.

**Conclusions:**

Results from a long and rigorous follow-up in a large cohort suggests that most MDD patients have an unfavourable prognosis. Longer follow-up and broader diagnostic conceptualisation show that the majority of patients have a disabling and chronic disorder. Conceptualising and handling MDD as a narrowly defined and episodic disorder may underestimate the prognosis of the majority of depressed patients and, consequently, the type of care that is appropriate.

**Electronic supplementary material:**

The online version of this article (doi:10.1186/s12916-017-0972-8) contains supplementary material, which is available to authorized users.

## Background

Major depressive disorder (MDD) has been historically perceived as an episodic disorder. In the early 1900s, Kraepelin differentiated between ‘dementia praecox’ (now known as schizophrenia), which he considered chronic and progressive, and ‘(manic) depression’, which he described as episodic [1, 2]. This view has ever since dominated our understanding of depression [3–5]. Congruently, longitudinal observational research over the past century suggests that most MDD patients eventually recover from their index episode after relatively short follow-up assessments (e.g. 2 years), and suggests that, ultimately, only a limited proportion follows a chronic course [6, 7]. This is further underlined by the fact that most intervention trial research has stringent treatment delivery and has focused on the short-term remission of an episode. Moreover, randomised controlled trials typically include a subset of less severe patients that have, for example, shorter illness duration and lower rates of anxious and atypical symptom features [8]. Their course outcomes might therefore not be representative and may be more positive than in ‘real world’ patients, which has resulted in the idea that the majority of patients recover over a relatively short time span and that only a minority has a chronic course. Although the clinical course of MDD has been an extensive topic of debate, research outcomes have generally given rise to an optimistic view. Moreover, this has translated into correspondingly optimistic communication with patients and into clinical management that aims for relatively short and episode-oriented treatments.

Although MDD may indeed be limited to a single episode in some patients, Judd et al. [9] have shown that most patients move in and out of more or less severe levels of symptoms over time. This suggests that, given a longer time frame, the prognosis is less favourable and that studies with a short follow-up time or relatively few assessments will tend to underestimate the prognosis of MDD. Further, although depression, bipolar disorder and anxiety disorders are conceptualised as distinct disorder groups, they are closely related in terms of genetics [10] and etiology, such as shared risk factors (e.g. childhood trauma and negative life events [11]) and similar physiological dysregulations [12]. Moreover, comorbidity levels are as high as 75% [13]; thus, taking into account anxiety disorders into the prognosis of MDD is extremely significant from the patients’ perspective. Finally, the three diagnostic constructs overlap in treatment outcomes and prognosis [14, 15]. Consequently, studies on the longitudinal course of MDD that do not take comorbidity into account might incline towards outcomes that are actually unrealistically favourable [16, 17]. Persons who have recovered from MDD and are labelled as such might still meet the full criteria of another, related psychiatric disorder and thus still suffer from marked functional impairment. It is therefore important, especially from the patients’ perspective, to consider the impact of other frequently co-occurring disorders, such as dysthymia, (hypo)mania and anxiety, when examining the full clinical course of MDD.

In this paper, using data from the Netherlands Study of Depression and Anxiety (NESDA), an on-going longitudinal psychiatric cohort study ideally designed to examine the long-term course of depressive and anxiety disorders [18], we test the idea that the course of patients with MDD changes seriously when different clinically meaningful course perspectives are considered. We examine the clinical course of MDD, expanding the perspective in terms of (1) a longer time frame and (2) a broader diagnostic conceptualisation including symptoms of closely related affective and anxiety disorders, thereby moving from a short, narrow course perspective to a long, broad course perspective. MDD might have a less favourable prognosis if one looks beyond diagnostic boundaries, which would warrant a change in our communication with patients and clinical management.

## Methods

### Study sample

Data were retrieved from NESDA [18], which, at baseline (2004–2007), consisted of 2981 persons aged between 18 and 65 years, recruited from community (19%), primary care (54%) and specialised mental healthcare (27%). The sampling frame was designed to represent the various developmental stages of depressive and anxiety disorders, and included patients with current or remitted depressive and/or anxiety disorder (74%) and healthy controls (26%). Exclusion criteria were (1) insufficient command of the Dutch language and (2) a primary clinical diagnosis of other severe psychiatric conditions such as bipolar disorder and schizophrenia. The study was approved by the Ethical Review Board of participating centres and all participants signed informed consent. Every 2 years, face-to-face follow-up assessments were conducted, with a response rate of 87.1% (*n* = 2596) at 2 years, 80.6% (n = 2402) at 4 years, and 75.7% (*n* = 2256) at 6 years. If participants missed an in-between follow-up assessment they were assessed over the time period since the last assessment.

At baseline, a diagnosis of MDD was assessed with the DSM-IV Composite International Diagnostic Interview (CIDI) version 2.1 [19]. A total of 1115 participants were diagnosed with an MDD episode, of any duration, during the 6 months prior to baseline (i.e. a 6-month diagnosis). Depressive symptoms in the week prior to assessment were measured with the Inventory of Depressive Symptomatology (IDS) self-report and were considered clinically relevant if the score was more than 13 [20].

The present study sample included 903 patients who had a current 6-month MDD diagnosis with clinically relevant depressive symptoms at baseline, and who had at least one follow-up assessment available. Compared to those with a 6-month MDD diagnosis that were excluded (total *n* = 212) because of low IDS score (*n* = 70) or no follow-up (*n* = 142), the included sample was older (*P* = 0.002), but was similar with respect to sex and years of education. Follow-up information was available for 903 participants at 2 years, 811 participants at 4 years and 712 participants at 6 years, providing an overall of 3329 observations.

### Sample descriptive characteristics

Age, sex and years of education were assessed during the baseline interview. Age at onset of MDD and whether the baseline episode was a first or recurrent MDD episode were derived from the CIDI. The severity of depressive and anxious symptoms in the week prior to assessment were examined using the IDS and the Beck Anxiety Inventory (BAI) – Self Report [21], respectively. Medications used in the month prior to baseline were registered according to the World Health Organization Anatomical Therapeutic Chemical classification [22]. Selective serotonin reuptake inhibitors (N06AB), tricyclic antidepressants (N06AA) and other antidepressants (N06A, not N06AA, not N06AB) were considered antidepressant treatment. Psychological treatment was considered received if at least three sessions provided by a healthcare professional were reported for the prior 6 months, as measured with the Trimbos/iMTA questionnaire for Costs associated with Psychiatric Illness [23]. Treatment was categorised as present (antidepressant and/or psychological treatment) or absent. A current comorbid diagnosis of dysthymia and/or anxiety disorder (social phobia, panic disorder, agoraphobia, generalised anxiety disorder (GAD)) was assessed with the CIDI [19].

### Course trajectories

Course trajectories were determined using two sources of data collected during each 2-year follow-up assessment, namely (1) the CIDI and (2) the Life Chart Interview (LCI) [24], and by joining data from all follow-up assessments into a continuous record. The CIDI determined the presence of DSM-IV classified MDD, dysthymia, (hypo)mania symptoms and anxiety disorders during each 2-year follow-up. For all patients with a depressive, (hypo)mania or anxiety disorder diagnosis according to the CIDI interview, information on the LCI was considered. The LCI uses a calendar method, wherein life-events were recalled to refresh memory, after which the presence of at least mild depressive, (hypo)mania, anxious and/or avoidance symptoms – separately – at each month during the 2-year follow-up was determined. The proportion of time with symptoms (=duration) was calculated for each 2-year interval by dividing the number of months with symptoms by the total number of months between two assessments. An episode was considered chronic if a person had a diagnosis on the CIDI in the 6 months prior to assessment and consistent symptoms for a minimum of 2 years (≥85% of time) on the LCI. If a person experienced only one disorder (e.g. MDD), the duration of only that disorder (e.g. depressive symptoms) was taken into account. If a person experienced multiple disorders (e.g. MDD and GAD), symptom duration of these disorders (e.g. depressive and/or anxiety symptoms) were integrated. Symptom durations acquired by the LCI were thus only considered in the presence of a CIDI-diagnosed disorder.

Subsequently, we classified four course trajectories describing the course at three time points (2-, 4- and 6-year follow-up):

1. Recovered: no diagnosis at 2-year follow-up or thereafter;
2. Recurrent, without chronic episodes: one or more diagnoses after baseline, but never a chronic episode;
3. Recurrent, with chronic episodes: one or more diagnoses after baseline and at least one chronic episode, but not at every follow-up assessment;
4. Consistently chronic: a diagnosis is consistently present, a chronic episode at every follow-up assessment.

These course trajectories were described from three diagnostic perspectives, namely (1) MDD only, (2) affective disorders (MDD, dysthymia and (hypo)mania) and (3) affective and anxiety disorders (MDD, dysthymia, (hypo)mania, panic disorder, agoraphobia, social phobia and GAD). In total, this resulted in a stepwise comparison of the prognosis, stepping up in time (three follow-up periods) and broadening the diagnostic conceptualisation by including affective and anxiety disorders (three diagnostic perspectives).

### Clinical validation characteristic

To test concurrent functional impairment we compared the level of disability of the four course trajectories at each assessment within the long, broad perspective (6-year follow-up, all affective and anxiety disorders, for *n* = 712 participants). Disability was measured with the World Health Organization Disability Assessment Schedule II (WHODAS-II) [25] at all assessments. We included all subscales except for the 4-item work disability (total 32 items) to avoid missing answers due to MDD patients often not working.

### Statistical analyses

Sociodemographic and clinical characteristics were reported as percentages, or means and standard deviations (SD). Occurrence of specific diagnoses, and the total time spent with depressive, anxious, avoidance and (hypo)mania symptoms, regardless of whether a CIDI diagnosis was present, between baseline and 2-year, baseline and 4-year, and baseline and 6-year follow-up were reported as percentages.

The proportion of patients categorised in the defined course trajectories were presented in nine different pie charts reflecting the time by diagnostic perspectives. As a clinical validation of the course trajectories, the longitudinal association between disability scores (WHODAS-II) and the four course trajectories according to the long, broad perspective (6-year follow-up, all affective and anxiety disorders; *n* = 712; observations = 2661) was estimated using a linear generalised estimating equations (GEE) model (with an exchangeable correlation structure), accounting for within person correlation of repeated measures and missing observations. The GEE model included a categorical time variable indexing the assessment wave and was additionally adjusted for baseline age, sex and years of education. Analysis was conducted using SPSS version 22.0 [26].

## Results

At baseline (Table 1), the mean age of the study sample was 41.4 years (SD = 12.0), 67% was female, and both the average scores on depressive and anxiety symptoms were indicative of moderate severity [27]. Additionally, more than two-thirds (70.9%) had at least one comorbid disorder at baseline.

**Table 1:** Descriptive characteristics of patients at baseline (*n* = 903)

Sociodemographics		
Age, years, mean (SD)		41.4 (12.0)
Female sex, % (n)		67.0 (605)
Education, years, mean (SD)		11.6 (3.2)
Clinical characteristics		
Age at onset of major depressive disorder, years, mean (SD)		27.3 (12.6)
Recurrent episodes, yes, % (*n*)		52.6 (475)
Severity of depressive symptoms (IDS), mean (SD)		33.9 (10.9)
Severity of anxiety symptoms (BAI), mean (SD)		18.6 (10.9)
Treatment (antidepressants and/or psychotherapy), % (*n*)		62.8 (567)
Recruitment setting	Community, % (n)	8 (72)
	Primary care, % (n)	37.5 (339)
	Specialised mental healthcare, % (*n*)	54.5 (492)
Current comorbidity^a^	Dysthymia, % (*n*)	24.1 (218)
	Generalised anxiety disorder, % (*n*)	30.2 (273)
	Social phobia, % (*n*)	35.5 (321)
	Panic disorder, % (*n*)	35.0 (316)
	Agoraphobia, % (*n*)	30.0 (271)
	Any other diagnoses, % (*n*)	70.9 (640)

^a^Current comorbidity = diagnosis present in 6 months prior to baseline; patients with comorbid disorders appear in more than one diagnosis group

*BAI* Beck Anxiety Inventory, *IDS* Inventory of Depressive Symptomatology, *n* number, *SD* Standard Deviation

During 2 years of follow-up (Table 2), the recurrence of MDD was 63.2% and 60.5% of patients had at least one other disorder. Over the entire 6 years of follow-up, the recurrence of MDD (after baseline) increased to 77.1% and 74.2% of participants had at least one other disorder.

**Table 2:** Occurrence of diagnoses after baseline at follow-up^a^

	0 to 2-year follow-up *n* = 903	0 to 4-year follow-up *n* = 811	0 to 6-year follow-up *n* = 712
Diagnoses	% (*n*)	% (*n*)	% (*n*)
MDD	63.2 (571)	72.3 (586)	77.1 (549)
Dysthymia	27.0 (244)	33.5 (272)	38.2 (272)
Generalised anxiety disorder	17.6 (159)	27.1 (220)	33.3 (237)
Social phobia	26.9 (243)	32.6 (264)	35.5 (253)
Panic disorder	22.4 (202)	28.1 (228)	32.2 (229)
Agoraphobia	21.3 (192)	26.0 (211)	28.2 (201)
(Hypo)manic symptoms	8.2 (74)	10.0 (81)	11.4 (81)
Any other diagnoses (excluding MDD)	60.5 (546)	69.3 (562)	74.2 (528)

^a^Patients with comorbid disorders appear in more than one diagnosis group

*MDD* major depressive disorder

Further, over the 6 years of follow-up, a substantial proportion of patients spent more than 75% of the time with depressive (19.4%), anxious (18.7%) or avoidance (10.3%) symptoms, while this proportion was considerably lower for (hypo)mania symptoms (0.1%) (Table 3).

**Table 3:** Percentage of time spent with symptoms during follow-up

	0–25% of time	25–50% of time	50–75% of time	75–100% of time
Symptoms	%	%	%	%
Percentage of time with symptoms during 0 to 2-year follow-up^a^(*n* = 903)				
Depressive symptoms	40.4	14.4	8.2	36.9
Anxiety symptoms	44.4	10.0	8.0	37.5
Avoidance symptoms	70.3	4.3	3.4	21.8
(Hypo)manic symptoms	98.1	0.3	0.2	1.3
Percentage of time with symptoms during 0 to 4-year follow-up^a^(*n* = 811)				
Depressive symptoms	38.1	18.0	20.1	23.2
Anxiety symptoms	35.9	17.8	21.6	24.3
Avoidance symptoms	61.4	8.0	17.4	12.7
(Hypo)manic symptoms	97.8	1.2	0.6	0.4
Percentage of time with symptoms during 0 to 6-year follow-up^a^(*n* = 712)				
Depressive symptoms	35.5	27.1	17.1	19.4
Anxiety symptoms	32.3	27.4	21.1	18.7
Avoidance symptoms	57.2	19.4	12.6	10.3
(Hypo)manic symptoms	97.6	1.7	0.6	0.1

^a^Time spent with symptoms regardless whether CIDI diagnoses present

### The course of MDD

**Fig. 1:**
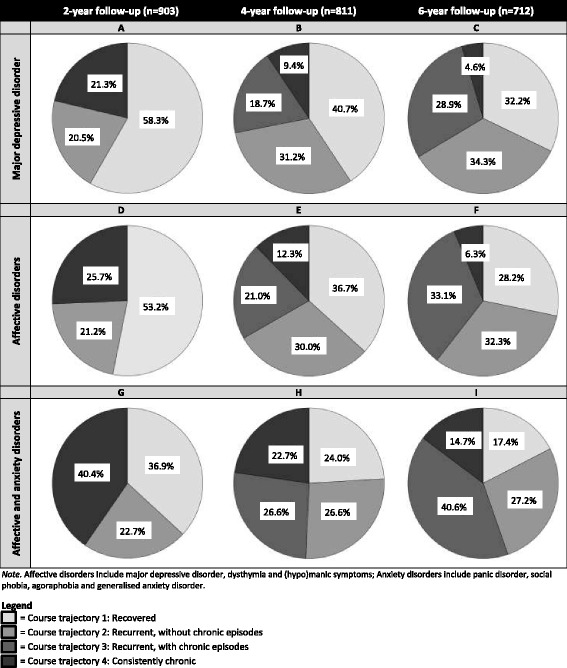
Pie charts of the course trajectories over 2-, 4- and 6-year follow-up for the different diagnostic categories

Figure 1 presents nine different pie charts showing the proportions of patients classified in the defined course trajectories for each of the three time points (columns) and each of the three diagnostic perspectives (rows).

#### To what extent does follow-up period impact the course of MDD?

The first row of Fig. 1 shows that, at 2-year follow-up, 58% of the baseline MDD patients had recovered from MDD, while 21% had recurrent MDD episodes and a further 21% had a chronic MDD episode (Fig. 1a). However, examining Fig. 1b and c shows that sustained recovery rates dropped to 41% and 32% at 4- and 6-year follow-up, respectively. Moreover, 34% (28.9 + 4.6) of patients had chronic MDD episodes of at least 2-years duration at the 6-year follow-up (Fig. 1c). A similar decrease in recovery rates and increase in proportion of patients with chronic episodes is observed in the second (Fig. 1d–f) and third rows (Fig. 1g–i).

#### To what extent does a broader diagnostic conceptualisation impact the course of MDD?

Examining the first column of Fig. 1 reveals that full recovery rates at 2-year follow-up decreased from 58% when considering only MDD (Fig. 1a) to 53% when including dysthymia and (hypo)mania symptoms (Fig. 1d). Only 37% of the baseline MDD patients remained fully recovered when additionally considering comorbid anxiety (Fig. 1g). A similar pattern was observed at 4- (Fig. 1b, e, h) and 6-year follow-up (Fig. 1c, f, i).

#### To what extent does the combination of both follow-up and broader diagnostic conceptualisation impact the course of MDD?

Examining only MDD over 2 years (the shortest, most narrow perspective; Fig. 1a) suggests a relatively benign prognosis, wherein 58% of patients recovered from MDD and 21% had a chronic episode. However, employing a longer time frame (6 years) and broadening the conceptualisation of affective and anxiety disorders (including dysthymia, (hypo)mania and anxiety disorders) (longest, broadest perspective, Fig. 1i) suggest a much less favourable prognosis, with only 17% of patients having early and sustained recovery and over 55% (40.6 + 14.7) with chronic episodes (Fig. 1i).

Additional file 1: Figure S1 showed that including only patients with complete data at all follow-up assessments (*n* = 712) did not change the above results as overall findings and percentages were very comparable.

### Clinical validity of course trajectories

**Fig. 2:**
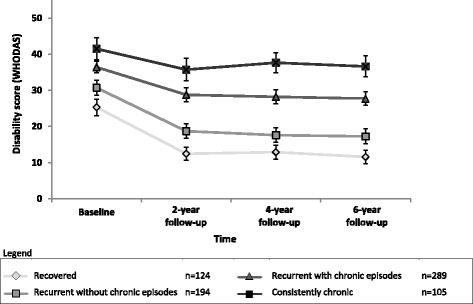
Mean disability scores (WHODAS) over time of the four course trajectories according to the longest, broadest perspective (6-year follow-up; all affective and anxiety disorders; Fig. 1i) (n = 712)

Figure 2 showed the raw mean disability (WHODAS-II) scores at baseline and at 2-, 4- and 6-year follow-up for the four course trajectories according to the long, broad perspective. Within all groups, the average disability was most severe at baseline, when all persons were in current episodes. GEE analysis adjusted for age, sex and years of education confirmed that, compared to those who recovered in the first 2 years and remained free of affective and anxiety disorder episodes until the 6-year follow-up, disability was consistently higher in patients with ‘recurrent without chronic episodes’ (B = 5.3, SE = 1.1, *P* < 0.001), in patients with ‘recurrent with chronic episodes’ (B = 14.1, SE = 1.0, *P* < 0.001) and in those with a ‘consistently chronic’ trajectory (B = 21.3, SE = 1.4, *P* < 0.001) over 6 years. This supports the clinical validity of the four course trajectories by indicating that the course groups not only differ in terms of symptoms but also in terms of general functioning.

## Discussion

This study tested the idea that the clinical course of patients with MDD may be underestimated when a narrow perspective is used with respect to either the time frame or the diagnostic conceptualisation. Considering a long and rigorous follow-up in a large cohort of patients with MDD reveals that we may need to reconsider our conceptualisation of MDD. Including symptoms of closely related disorders, such as (hypo)mania and anxiety symptoms, shows that the majority of patients have a disabling and chronic depressive disorder. Conceptualising MDD as a narrowly defined and episodic disorder may underestimate both the prognosis for the majority of our patients and, consequently, the type of care that is appropriate. With a short, narrow perspective (2-year follow-up and MDD only), 58% of patients appeared recovered and only a minority (21%) had a chronic episode. With a long, broad perspective (6-year follow-up, including affective and anxiety disorders) the recovery rate decreased to 17% and the proportion of patients with chronic episodes increased to 55%. The impact on the daily functioning was found to be parallel to the severity of the course trajectory.

The current findings suggest that we may need to rethink the conceptualisation of depression from an episodic and isolated disorder to a recurrent and often chronic disorder with high levels of comorbidity. Since the nineteenth century, depression has been characterised in numerous ways. In 1883, Clouston was one of the first to describe melancholia with its symptoms [28]. This was further elaborated by Kraepelin [28, 29], who advocated more emphasis on prognosis, functional consequences or aetiology. Although DSM-5 [30] has become more sensitive to both the development of affective disorders over time and the inclusion of symptoms of co-occurring disorders, healthcare systems and treatment protocols generally still conceptualise MDD as relatively short-term and episodic. Moreover, lay people tend to view depression as an incident in response to life stress rather than a disorder that is often chronic [31]. Additionally, several studies on the prospective long-term course of depression in the general population and primary care describe high rates of stable recovery from depressive symptoms (35–60%) [15]. The current study, however, found that recovery rates were considerably lower when relevant co-morbidity is included. Consequently, this suggests that approaching depression as a recurrent, but mostly ‘time-limited’, episodic disorder may amount to an underestimation of its severity and clinical burden. We showed that only a minority of depressed patients experienced early and sustained recovery from all affective and anxiety conditions, while the majority experienced a pattern of recurrent and often chronic episodes. The long-term impact that such clinical courses have on a person’s level of functioning was confirmed by our clinical validation, where patients with chronic episodes had consistently higher disability levels compared to those without chronic episodes and those that recovered.

Currently, psychiatric disorders are strictly categorised to limit overlap and increase specificity. However, in a recent review paper [32], Kendler showed that the large majority of descriptions of major depression up until the 1960s included anxiety as a symptom of depression. In line with this, our results suggest that depression and anxiety are not entirely separate constructs. The high levels of co-occurrence [13, 33], overlapping etiologies [10, 12] and similar treatments that are effective for depression and anxiety further support this. Our data suggest that including co-occurring anxiety yields a more valid, albeit less favourable, picture of the prognosis of depression. As in other areas in medicine, where the prognosis of disorders is often chronic, such as in rheumatoid arthritis, diabetes or chronic lung disorders, we may need to shift our attention more to the functional consequences of disorders when conceptualising the prognosis and testing interventions. Using the DSM-5 instrument for functioning, our data suggest that a broader and longer-term perspective of the prognosis of affective disorders is ecologically and clinically relevant. Our findings suggest that clinicians should consider interventions aimed at treating MDD as a chronic disorder and should more systematically incorporate relapse prevention strategies. Treatment should further focus on recovery from multiple symptom domains (including, for example, anxiety and hypomania) as well as functional recovery. Moreover, clinical trials should aim to include a representative and ecologically valid study sample [8].

Our study is unique in that we have access to a large cohort of patients representing the full range of depression and anxiety disorders. Patients were rigorously diagnosed and followed up over a longer period of time. Information was available throughout the 6-year follow-up period, allowing us to reliably categorise different course trajectories. However, some limitations of this study should also be noted. First, categorisation of the heterogeneous prognosis into four course trajectories inevitably results in arbitrary decisions for some cases, especially those with missing information on the life-chart. However, we showed that exclusion of those with missing information did not change the results substantially (Additional file 1: Figure S1)*.* In our definition of the course trajectories, duration of (mild) symptomatology was not considered without the presence of a full diagnosis. Hence, this definition of the course trajectories might have underestimated the chronicity. Second, information on comorbidity was considered for a limited number of disorders. It would have been informative to further include symptomatology of other psychiatric disorders (e.g. obsessive compulsive disorder or substance use disorders) into account. This may even further impact on an unfavourable prognosis, but is beyond the focus of our current paper in which we decided on the most common affective and anxiety disorders that are closely linked with MDD in terms of overlap in etiology, symptomatology and treatment indications. Further, a subset of our sample was aged above 60 years (18.7% at 6-year follow-up) and consequently may have somatic morbidity, which could have attenuated the likelihood of sustained recovery. Third, it should be noted that the observational design of our study is not ideal to draw conclusions regarding treatment. This study does not address the impact of specific treatments on the prognosis of MDD. It would, naturally, be of great importance for future research to examine whether specific and carefully delivered treatment could impact outcomes more profoundly. Nevertheless, our results do show that, despite the fact that the mental healthcare system in the Netherlands is accessible, affordable and of good quality [34], outcomes are generally unfavourable. Fourth, the NESDA study commenced (2004) prior to the appearance of DSM-5, and therefore diagnoses in this paper reflect DSM-IV categories – of which some are no longer present in DSM-5 (e.g. dysthymia). Finally, during follow-up, there was selective loss of patients. Since those who are lost to follow-up are generally the worst affected cases, selective loss tends to decrease the number of cases that would be classified as chronic at later follow-up; therefore, our results might underestimate the severity of the course.

## Conclusion

Our data suggest that we may need to reconsider the prognosis of patients with MDD. Including symptoms of closely related disorders, such as (hypo)mania and anxiety, shows that the majority of patients have a disabling and chronic affective disorder and that full recovery is the exception rather than the rule. Conceptualising MDD as a narrowly defined and episodic disorder may underestimate both the prognosis of the majority of our patients and the type of care that is appropriate.

## Additional file

Additional file 1:
**Figure S1.** Pie charts patients with complete follow up data. (DOCX 243 kb)

## Abbreviations

BAI: Beck Anxiety Inventory
CIDI: Composite International Diagnostic Interview
DSM: Diagnostic and Statistical Manual of Mental Disorders
GAD: Generalised Anxiety Disorder
GEE: Generalised Estimating Equations
IDS: Inventory of Depressive Symptomatology
LCI: Life Chart Interview
MDD: Major Depressive Disorder
NESDA: Netherlands Study of Depression and Anxiety
WHODAS-II: World Health Organization Disability Assessment Schedule II.
