# Supplementary material for: Reconsidering the prognosis of major depressive disorder across diagnostic boundaries: full recovery is the exception rather than the rule
Source: BMC Med. 2017 Dec 12;15:215. doi: 10.1186/s12916-017-0972-8 (PMC5725897; doi:10.1186/s12916-017-0972-8)
Supplement: Additional file 1: — Figure S1. Pie charts patients with complete follow up data. (DOCX 243 kb) [file 12916_2017_972_MOESM1_ESM.docx]

**Additional file 1: Figure S1.** Pie charts of the course trajectories over 2-year, 4-year and 6-year follow-up for the different diagnostic categories for patients with complete data at all follow-up assessments (n=712)

|  | **2-year follow-up (n=712)** | **4-year follow-up (n=712)** | **6-year follow-up (n=712)** |
| --- | --- | --- | --- |
|  | **A** | **B** | **C** |
| **Major depressive disorder** | 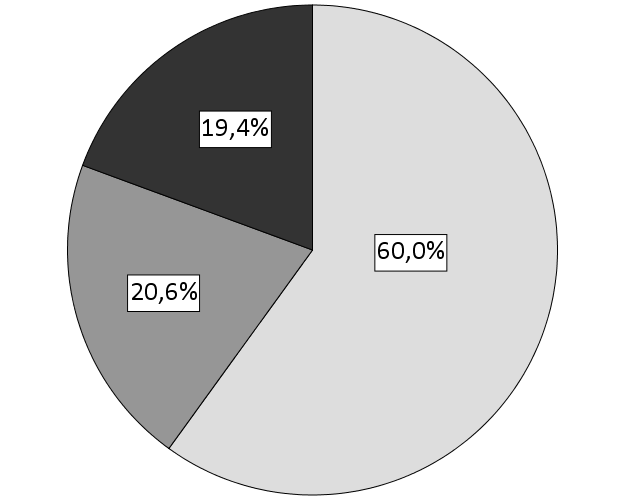  20.6%  60.0%  19.4% | 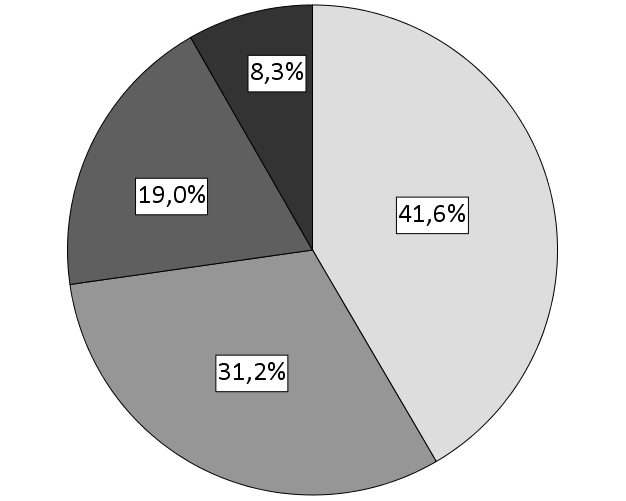  8.3%  19.0%  31.2%  41.6% | 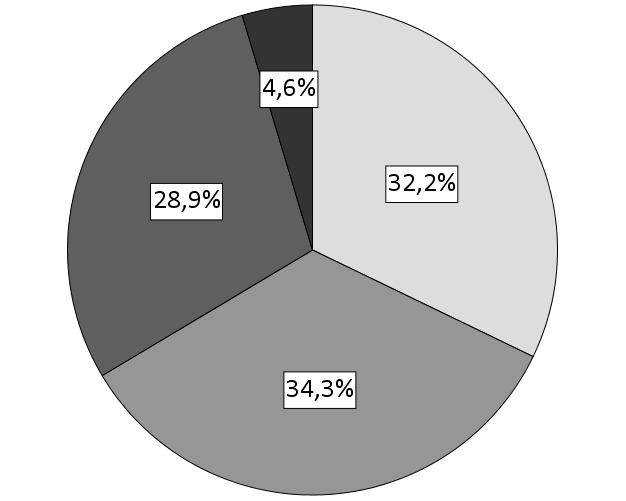  28.9%  34.3%  32.2%  4.6% |
|  | **D** | **E** | **F** |
| **Affective disorders** | 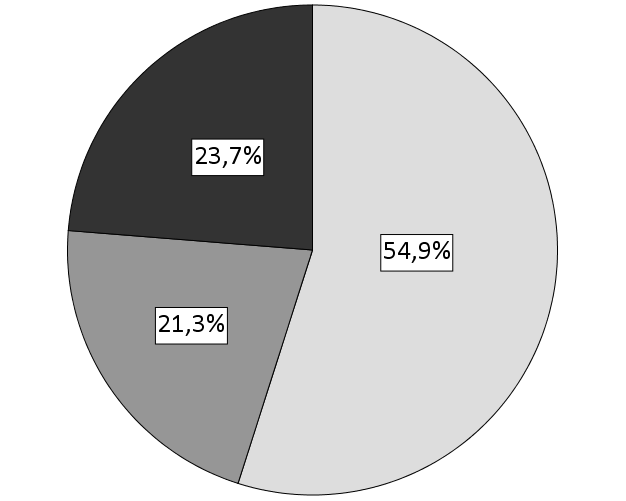  54.9%  21.3%  23.7% | 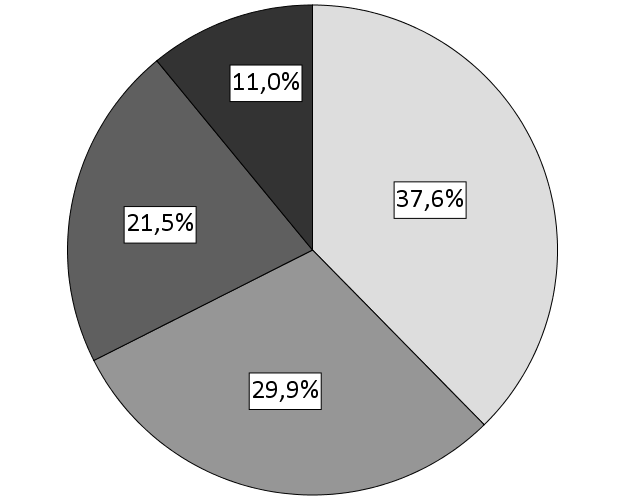  29.9%  11.0%  21.5%  37.6% | 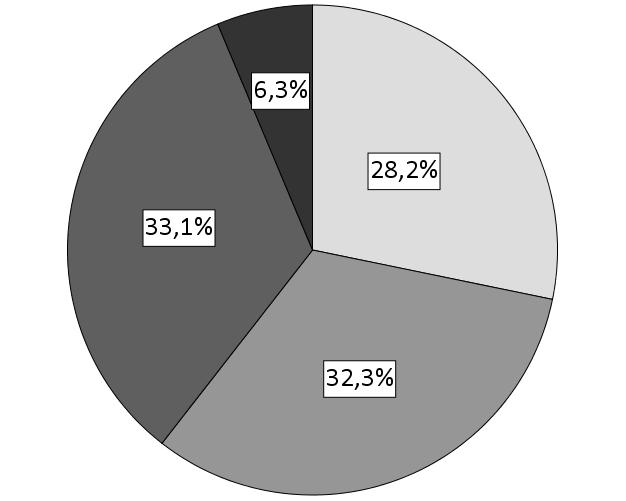  6.3%  33.1%  32.3%  28.2% |
|  | **G** | **H** | **I** |
| **Affective and anxiety disorders** | 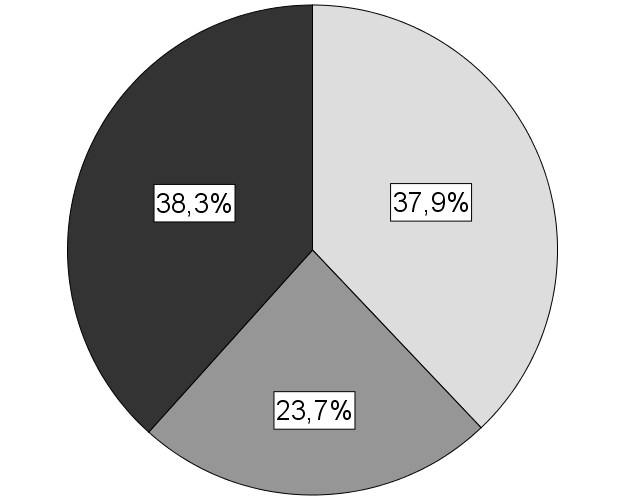  23.7%  38.3%  37.9% | 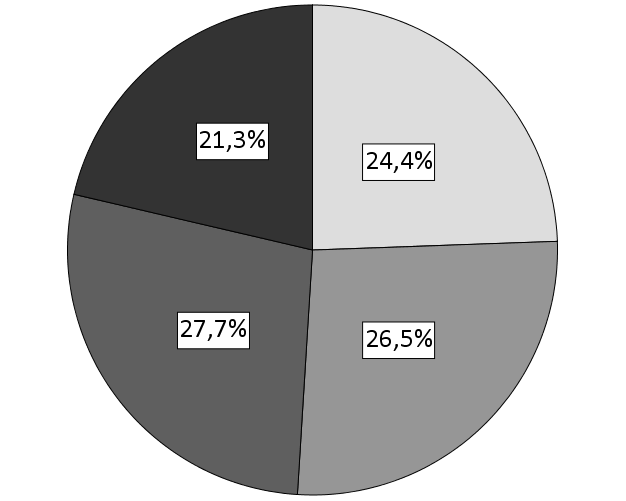  27.7%  21.3%  24.4%  26.5% | 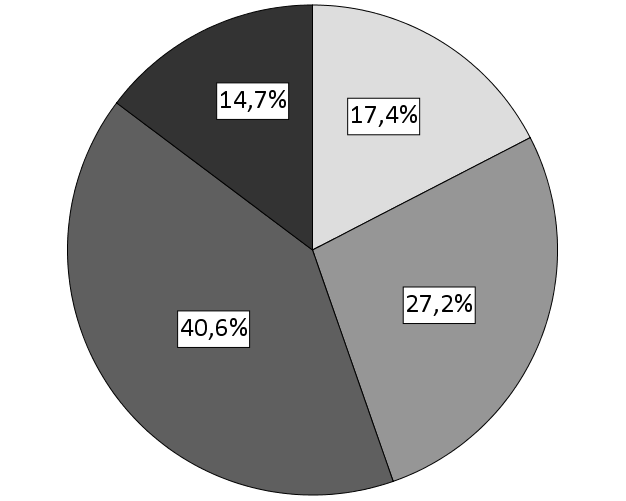  14.7%  17.4%  40.6%  27.2% |

*Note.* Affective disorders include major depressive disorder, dysthymia and (hypo)manic symptoms; Anxiety disorders include panic disorder, social phobia, agoraphobia and generalised anxiety disorder.

**Legend**

= Course trajectory 1: Sustained recovery
 = Course trajectory 2: Recurrent, non-chronic
 = Course trajectory 3: Recurrent, chronic
 = Course trajectory 4: Consistently chronic
